# Evaluation of joint external evaluation to COVID-19 and other infectious diseases mortality outcomes in 96 countries

**DOI:** 10.1093/inthealth/ihae077

**Published:** 2024-12-04

**Authors:** Yuri Lee, Siwoo Kim, Sieun Lee, Min Kyung Kim, Lawrence O Gostin, Juhwan Oh

**Affiliations:** Department of Health and Medical Information, Myongji College, Seodaemun-Gu, Seoul 03656, Republic of Korea; Division of Disease Surveillance Strategy, Korea Disease Control and Prevention Agency, 187 Osongsaengmyeong2-ro, Osong-eup, Heungdeok-gu, Cheongju-si, Chungcheongbuk-do, 28159, Republic of Korea; Department of Health and Medical Information, Myongji College, Seodaemun-Gu, Seoul 03656, Republic of Korea; Gillings School of Global Public Health, Carolina Population Center, University of North Carolina at Chapel Hill, 135 Dauer Dr., Chapel Hill, NC 27599, USA; O'Neill Institute for National and Global Health Law, Georgetown University Law Center, 600 New Jersey Ave., NW Washington D.C. 20001, USA; Department of Medicine, Seoul National University College of Medicine, 103 Daehak-ro, Jongno District, Seoul 03087, Republic of Korea

**Keywords:** COVID-19, infectious diseases, Joint External Evaluation

## Abstract

**Background:**

This study evaluated the effectiveness of Joint External Evaluation (JEE) scores with regard to coronavirus disease 2019 (COVID-19) and other infectious diseases performance in 96 countries. To propose a revised JEE tool, potential JEE indicators were also examined.

**Methods:**

JEE data from 2016–2019 were linked with outcomes such as COVID-19 fatality rates and infections, as well as mortality rates for other infectious diseases. We also examined potential indicators such as the Sustainable Development Goals (SDGs) and Universal Health Coverage index to propose enhancements to the JEE tool. Multiple regression analysis was used to assess these associations.

**Results:**

The average JEE score was 2.70 (SD=0.92) in 96 countries. Detection capabilities received the highest average score (3.23), while the other areas (2.30) section received the lowest scores. However, the analysis revealed that the JEE tool had limited predictive accuracy for COVID-19 outcomes. By contrast, the JEE scores showed a negative association with the performance of other infectious diseases. Notably, SDGs 2 (zero hunger), 4 (quality education) and 8 (decent work and economic growth) were strongly associated with better COVID-19 outcomes.

**Conclusion:**

The JEE scores showed limited predictive value for COVID-19 mortality outcomes in 96 countries. The tool offers insights into health security, but needs revision to better handle future pandemics.

## Introduction

On 30 January 2020, the WHO declared coronavirus disease 2019 (COVID-19) a public health emergency of international concern.^1^ By the time the WHO ended the public health emergency on 5 May 2023, there had been >6 million deaths reported. Years later, >370 million confirmed cases and >5.6 million deaths have been reported globally.^2^ This global health crisis revealed significant weaknesses in public health systems. Mistrust of scientific evidence, underinvestment in healthcare systems and poor surveillance systems left many countries unprepared to respond to the COVID-19 pandemic.^3^ These inadequate responses should be important lessons for countries to re-evaluate their health security status for future pandemics. These inadequate responses highlight the urgent need for countries to re-evaluate and strengthen their health security measures to better prepare for future pandemics.

One of the tools developed to assess and improve a country's preparedness for health emergencies is the Joint External Evaluation (JEE). The JEE evaluates a country’s capacity to prevent, detect and respond to public health threats like pandemics.^4^ The JEE was developed as a tool to evaluate core health system capacities under the International Health Regulations (IHR) in 2016.^5^ In the context of COVID-19, understanding how well countries were prepared according to JEE scores, and comparing those with their actual performance during the pandemic, provides valuable insights into the effectiveness and limitations of the JEE as a predictive tool. By the end of 2021, more than one-half of all WHO member states (113 countries) had conducted a JEE. The JEE assesses several critical areas, including prevention, detection and response capabilities, providing a comprehensive view of a nation's preparedness for health emergencies. In 2016, the average JEE score was 82% and 46% in the USA and Bangladesh, respectively.^5^ However, the significant disparity in COVID-19 outcomes, with deaths per million people being four times higher in the USA than in Bangladesh, raises questions about the predictive validity of JEE scores. This discrepancy may be influenced by various factors, including differences in healthcare infrastructure, population demographics and policy responses. For example, the USA has a higher proportion of older people and a greater prevalence of comorbidities, which are risk factors for severe COVID-19 outcomes. Moreover, differences in governmental policy responses, such as the timing and strictness of lockdowns, testing availability and public health messaging, likely played a significant role in these outcomes.^6^ This suggests that while the JEE provides important insights into a country's preparedness, other factors not captured by the JEE may also critically determine pandemic outcomes.

Previous research, including a study by Haider et al., has found that the JEE had a low predictive value for early COVID-19 case detections and mortality rates across 168 countries.^7^ This discrepancy suggests that the JEE may not fully capture the complexities of pandemic preparedness and response, particularly in the context of a novel virus like COVID-19. The contrast between expected outcomes based on JEE scores and actual COVID-19 mortality underscores the need to reassess the JEE tool's effectiveness in its current form.

Given the critical role of the JEE in guiding global health security efforts, it is essential to investigate its actual predictive value, particularly in the context of COVID-19. Understanding whether JEE scores truly reflect a country's capacity to manage pandemics is crucial for future health security planning. In this paper, we estimated the association between JEE scores and COVID-19 performance across 96 countries. To provide a broader context, we repeated the same analysis for other infectious diseases performance. Furthermore, we examined potential indicators, such as Sustainable Development Goals (SDGs) and the Universal Health Coverage (UHC) index, to propose guidance for the development of a revised JEE tool that could more accurately predict and improve preparedness for future pandemics.

## Methods

### Data sources

For JEE scores, we collected data from the WHO Strategic Partnership Portal.^8^ The purpose of this portal is to monitor the health security capacity of WHO member states where detailed national profiles (including JEE scores) are provided. We collected the latest JEE scores available from 2016 to 2019 in 96 countries, selected from the 194 WHO member states, based on the availability of JEE data as of 9 July 2021.

For COVID-19 performance, the number of cases (cumulative count of detected and laboratory-confirmed positive cases), the number of deaths (cumulative number of deaths among detected cases) and the number of deaths per 1 million population were extracted from Worldometer as of 9 July 2021. The Worldometer uses official data sources like Ministry of Health reports and other government institutions and government authorities’ social media accounts to provide daily updated COVID-19 situation reports worldwide. Details of the data source for each country can be found elsewhere.^9^

For other infectious diseases performance, influenza and pneumonia deaths per 100 000 people (age standardized) in 2018 were extracted from World Health Rankings.^10^ All other indicator data were collected from World Health Statistics 2020, which reports the most recent statistics for the WHO member states.^11^

For potential JEE indicators, we collected the data in 2021 from the official websites of international organizations such as the United Nations, World Bank, United Nations Human Rights Office of the High Commissioner, Organization for Economic Cooperation and Development, Sustainable Development Solution Network, the Global Economy, Trading Economics, Family Health International, United States Agency for International Development, International Center for Not-for-profit Law and World Factbook Urbanization.

For the two covariates, data for the proportion of the population aged ≥65 y out of the total population in 2019 were collected from World Bank.^12^ The rate of urbanization in 2020 data were collected from World Factbook.^13^

### Measures

#### JEE score

The JEE tool consists of 49 indicators within 19 technical areas and these are organized into four groups: prevent, detect, response and other areas (IHR-related hazards and points of entry). Each indicator receives a score that ranges from 1 to 5: (1) no capacity; (2) limited capacity; (3) developed capacity; (4) demonstrated capacity; and (5) sustainable capacity. For this study we used JEE scores: overall, prevention, detection and response, based on the data available for the 96 countries included in the analysis.

#### COVID-19 performance

The COVID-19 fatality rate was calculated as the proportion of the number of deaths out of the number of cases. The number of COVID-19 infections per million was calculated as the number of cases per population in millions. The number of COVID-19 deaths per million was calculated as the number of deaths per population in millions.

#### Infectious diseases performance

For infectious diseases performance, we used nine measures: (i) new HIV infections per 1000 uninfected population; (ii) TB incidence per 100 000 population; (iii) malaria incidence rate per 1000 population at risk; (iv) hepatitis B surface antigen (HBsAg) prevalence among children aged <5 y (%); (v) diphtheria-tetanus-pertussis (DTP3) immunization coverage among 1-y-olds (%); (vi) measles-containing vaccine second-dose (MCV2) immunization coverage by the nationally recommended age (%); (vii) pneumococcal conjugate third-dose (PCV3) immunization coverage among 1-y-olds (%); (viii) HPV immunization coverage estimates among 15-y-old girls (%); and (ix) influenza and pneumonia deaths per 100 000 people.

#### Potential indicators

We divided the potential indicators into two groups. The first group includes trade, economic freedom (score out of 10), civil society participation, ratification of 18 International Human Rights Treaties, current health expenditure (% of gross domestic product), domestic general government health expenditure as the percentage of general government expenditure, trust in government, trust in government (confidence in police total), trust in government (trust worthiness and confidence), trust in government (public trust in politicians), political stability index (by the Global Economy), financial globalization indices, growth rates of household expenditure or income per capita, the proportion of people living below 50% of median income, the proportion of population reporting having felt discriminated against on the grounds of discrimination, gender and disability, social security rate and 2019 Civil Society Organization Sustainability Index.

The second group includes the SDG 1–17 indicators and UHC index, catastrophic health expenditure (exceeding 10% of total consumption expenditure) and catastrophic health expenditure (exceeding 25% of total consumption expenditure).

#### Covariates

The proportion of the population aged ≥65 y out of the total population and the urbanization rate were included as two covariates.

### Statistical analysis

First, multiple regression analyses were performed to determine the association between JEE scores and three COVID-19 performance measures (i.e. fatality rate, infections per million and deaths per million), adjusted by covariates. The multiple regression models assumed linear relationships between the independent variables (JEE scores, covariates) and the dependent variables (COVID-19 performance measures). Additionally, the models assumed that the residuals were normally distributed, homoscedastic and independent of each other. Tests for normality, multicollinearity and homoscedasticity were conducted to ensure the validity of these assumptions. Second, we repeated the first regression model with the other infectious diseases performance. Third, the COVID-19 performance and influenza and pandemic mortality rate were regressed to the potential JEE indicators. Lastly, we repeated the third regression model with the SDGs and UHC indicators. Statistical significance was determined as p<0.05, p<0.01 and p<0.001 in a two-sided manner. All statistical analysis was performed with SPSS 25.0 (SPSS Inc, Chicago, IL, USA), with a sample size of 96 countries, based on the availability of JEE data.

## Results

The average JEE score across 96 countries was 2.70 (SD=0.92) (Table 1). The highest JEE score was observed in the detection category (3.23), while the lowest score was found in the other areas category (2.30) (Table 1). The number of COVID-19 infections per million was 256.84 (434.54) (Table 1). Among other infectious diseases performance, the new HIV infection rate per 1000 uninfected population was the smallest at 0.85 (1.62) (Table 1). The overall SDGs average score was 60.60 (10.10) (Table 1).

**Table 1.** Summary of measures across 96 countries

Measures	Minimum	Maximum	Mean	SD
**JEE** **^a^**				
Overall score	1.36	4.60	2.70	0.92
Prevention	1.00	5.00	2.75	1.10
Detection	1.00	5.00	3.23	0.89
Response	1.07	4.93	2.59	1.06
Other areas	1.00	5.00	2.30	1.20
**COVID-19 performance** **^b^**				
COVID-19 fatality rate	6.00	94 115.00	16 036.88	23 844.31
Number of COVID-19 infections per million	0.20	1915.00	256.84	434.54
Number of COVID-19 deaths per million	3324.00	3 233 165.00	276 323.48	466 535.29
**Infectious diseases performance** **^c^**				
Influenza and pneumonia deaths (per 100 000 people)	2.05	246.59	69.29	56.64
New HIV infection (per 1000 uninfected population)	0.01	8.62	0.85	1.62
TB incidence (per 100 000 population)	0.00	486.50	145.38	144.69
Malaria incidence (per 1000 population at risk)	0.04	21.13	2.09	2.85
HBsAG prevalence among children aged <5 y (%)	41.00	99.00	86.66	13.75
DTP3 immunization coverage among 1-y-olds (%)	4.00	99.00	78.58	22.58
PCV3 immunization coverage among 1-y-olds (%)	3.00	99.00	81.25	18.05
HPV immunization coverage among 15-y-old girls (%)	0.00	99.00	51.80	28.20
**SDGs and UHC indicators** **^d^**				
Overall SDGs (Global Index Score)	37.70	83.00	60.60	10.10
Mean of overall SDGs except SDGs 3	38.65	82.15	60.45	9.60
Goal 1. No poverty	0.00	100.00	78.36	28.69
Goal 2. Zero hunger	14.70	83.20	50.00	13.06
Goal 3. Good health and well-being	21.80	96.70	62.96	20.06
Goal 4. Quality education	4.80	99.30	62.23	26.60
Goal 5. Gender equality	24.60	89.40	59.43	16.15
Goal 6. Clean water and sanitation	28.90	98.30	68.43	17.32
Goal 7. Affordable and clean energy	0.00	94.20	53.26	30.72
Goal 8. Decent work and economic growth	21.90	92.70	60.94	16.32
Goal 9. Industry, innovation and infrastructure	0.80	92.80	29.36	23.78
Goal 10. Reduce inequalities	0.00	100.00	56.31	23.05
Goal 11. Sustainable cities and communities	26.40	97.30	65.00	16.20
Goal 12. Responsible consumption and production	28.90	93.70	71.94	12.48
Goal 13. Climate action	23.30	95.80	80.14	13.90
Goal 14. Life below water	10.70	65.20	48.04	9.28
Goal 15. Life on land	25.70	90.40	60.35	12.35
Goal 16. Peace, justice and strong institutions	36.70	92.90	63.04	13.17
Goal 17. Partnerships for the goals	0.00	100.00	60.38	14.24
3.8. UHC service coverage index	25.00	89.00	58.01	16.53
3.8. Catastrophic health expenditure (exceeding 10% of total consumption expenditure)	0.20	544.20	9.05	9.04
3.8. Catastrophic health expenditure (exceeding 25% of total consumption expenditure)	0.10	22.20	2.40	3.47
**Potential JEE indicators**				
Trade	321.00	10.00	81.35	51.80
Median per capita income	15 725.00	47.00	3069.33	4627.51
Economic Freedom (score out of 10)	8.71	4.45	6.68	0.92
Civil society participation	9.00	1.00	4.59	1.88
Ratification of 18 International Human Rights Treaties	13.00	1.00	3.76	2.53
Current health expenditure (% of GDP)	16.89	2.14	6.15	2.87
Domestic general government health expenditure as percentage of general government expenditure	23.60	1.80	8.83	4.84
Trust in government	84.63	17.15	51.43	19.83
Trust in government (confidence in police total)	7.62	4.52	6.28	0.84
Trust in government (trust worthiness and confidence)	6.46	2.27	4.17	0.83
Trust in government (public trust in politicians)	6.42	1.59	3.37	1.19
Political stability index (by the Global Economy)	1.53	−0.27	0.42	0.52
Financial globalization indices	0.70	0.00	0.20	0.22
Growth rates of household expenditure or income per capita	8.00	−4.00	2.08	2.52
Proportion of people living below 50% of median income	25.00	4.00	13.33	5.34
Proportion of population reporting having felt discriminated against, on the grounds of discrimination, gender and disability	28.30	5.40	19.68	6.66
Social security rate	38.20	0.00	19.24	9.52
2019 Civil Society Organization Sustainability Index	5.70	2.50	4.41	0.70
**Covariate** **^e^**				
Population aged 65 y and older (% of total population)	1.00	28.00	7.30	6.43
Urbanization rate	−1.23	5.43	2.41	1.50

COVID-19: coronavirus disease 2019; DTP3: diphtheria-tetanus-pertussis; HBsAG: hepatitis B surface antigen; GDP: gross domestic product; JEE: Joint External Evaluation; PCV3: pneumococcal conjugate third dose; SDG: Sustainable Development Goal; UHC: Universal Health Coverage.

^a^JEE scores were collected from the WHO Strategic Partnership Portal from 2016 to 2019.

^b^COVID-19 performance data were collected from Worldometer as of 9 July 2021.

^c^Infectious diseases performance data were collected from World Health Rankings 2018 and World Health Statistics 2021.

^d^SDG and UHC data were collected from the Sustainable Development Solution Network and United Nations.

^e^Covariate data were collected from World Bank 2019 and World Factbook 2020.

The JEE score was positively associated with the number of COVID-19 infections per million (*β*=0.383, p=0.016) after adjusting with covariates (Table 2). This indicates that for each unit increase in the JEE score, the number of COVID-19 infections per million is expected to increase by approximately 38.3%. This positive association suggests that higher JEE scores, which indicate better national preparedness, may correlate with higher reported COVID-19 infection rates due to more comprehensive testing and reporting capabilities in countries with robust health infrastructures. The response and other areas sections of the JEE tool were also positively associated with the infection rate and the number of COVID-19 deaths per million (Table 2). These findings could indicate that countries with higher JEE scores were more capable of identifying and reporting COVID-19 cases and deaths, reflecting the strengths of their surveillance and healthcare systems, rather than a failure in their public health responses. However, the fatality rate of COVID-19 was not associated with the JEE scores (Table 2). These coefficients suggest that higher preparedness in these areas correlates with higher reported COVID-19 cases and deaths, potentially due to increased detection and reporting capabilities.

**Table 2.** Adjusted association between JEE scores and COVID-19 performance across 96 countries

	COVID-19 fatality rate^a^		Number of COVID-19 infections per million^b^		Number of COVID-19 deaths per million^a^	
JEE scores	*β*	p-value	*β*	p-value	*β*	p-value
JEE score^c^	−0.151	0.418	0.338*	0.016	0.275	0.137
Prevention	−0.086	0.663	0.338	0.021	0.213	0.282
Detection	−0.109	0.503	0.176	0.169	0.101	0.538
Response	−0.158	0.370	0.303*	0.027	0.197	0.267
Other areas	−0.297	0.120	0.348*	0.016	0.383*	0.042

COVID-19: coronavirus disease 2019; JEE: Joint External Evaluation.

*p<0.05.

^a^Adjusted by the proportion of population aged 65 y and older (% of total population) and urbanization rate.

^b^Adjusted by urbanization rate. The JEE scores were collected from the WHO Strategic Partnership Portal from 2016 to 2019. The JEE score ranges from 1 (no capacity) to 5 (substantial capacity) across 48 indicators. These indicators are divided into four sections: prevention, detection, response and other areas. We used the average score for each of the four sections and the overall average score of the four sections as the final JEE score^c^. For COVID-19 performance, the number of cases (cumulative count of detected and laboratory-confirmed positive cases), the number of deaths (cumulative number of deaths among detected cases) and the number of deaths per 1 million population were extracted from Worldometer as of 9 July 2021. The COVID-19 fatality rate was calculated as the proportion of the number of deaths out of the number of cases. The number of COVID-19 infections per million was calculated as the number of cases per population in millions. The number of COVID-19 deaths per million was calculated as the number of deaths per population in millions.

For other infection diseases performance, JEE scores showed an inverse association with influenza and pneumonia deaths per 100 000 people (*β*=−0.315, p=0.016), with similar negative trends across all four sections: prevention, detection, response and other areas (Table 3). Meanwhile, MCV2 and PCV3 immunization coverage were positively associated with JEE scores at 0.506 and 0.252, respectively (Table 3). New HIV infection rate, HBsAG prevalence, DPT3 and HPV immunization coverage showed no significant association with JEE scores (Table 3).

**Table 3.** Association between JEE scores and other infectious diseases performance across 96 countries

Other infectious diseases performance	JEE scores	*β*	p-value
Influenza and pneumonia deaths (per 100 000 people)^a^	JEE score^c^	−0.315*	0.016
	Prevention	−0.397**	0.004
	Detection	−0.241*	0.037
	Response	−0.276*	0.026
	Other areas	−0.278*	0.039
New HIV infection (per 1000 uninfected population)^b^	JEE score^c^	−0.118	0.307
	Prevention	−0.075	0.517
	Detection	−0.151	0.190
	Response	−0.114	0.323
	Other areas	−0.123	0.285
TB incidence (per 100 000 population)^b^	JEE score^c^	−0.186	0.071
	Prevention	−0.167	0.105
	Detection	−0.197	0.056
	Response	−0.221*	0.031
	Other areas	−0.148	0.153
Malaria incidence (per 1000 population at risk)^b^	JEE score^c^	−0.281*	0.038
	Prevention	−0.239	0.079
	Detection	−0.347*	0.010
	Response	−0.221	0.105
	Other areas	−0.326*	0.015
HBsAG prevalence among children aged <5 y (%)^b^	JEE score^c^	−0.078	0.450
	Prevention	−0.091	0.382
	Detection	−0.073	0.480
	Response	−0.075	0.469
	Other areas	−0.085	0.414
DTP3 immunization coverage among 1-y-olds (%)^b^	JEE score^c^	−0.037	0.723
	Prevention	−0.044	0.675
	Detection	−0.052	0.617
	Response	−0.007	0.943
	Other areas	−0.073	0.481
MCV2 immunization coverage by the nationally recommended age (%)^b^	JEE score^c^	0.506***	<0.001
	Prevention	0.542***	<0.001
	Detection	0.461***	<0.001
	Response	0.494***	<0.001
	Other areas	0.510***	<0.001
PCV3 immunization coverage among 1-y-olds (%)^b^	JEE score^c^	0.252*	0.025
	Prevention	0.234*	0.038
	Detection	0.205	0.070
	Response	0.284*	0.011
	Other areas	0.205	0.070
HPV immunization coverage among 15-y-old girls (%)^b^	JEE score^c^	−0.117	0.502
	Prevention	−0.023	0.894
	Detection	−0.028	0.872
	Response	−0.026	0.883
	Other areas	−0.115	0.512

DTP3: diphtheria-tetanus-pertussis; HBsAG: hepatitis B surface antigen; JEE: Joint External Evaluation; MCV2: Measles-containing vaccine second dose; PCV3: pneumococcal conjugate third dose.

*p<0.05.

**p<0.01.

***p<0.001.

^a^Data were collected from World Health Rankings in 2018.

^b^Data were collected from World Health Statistics in 2020. The JEE scores were collected from the WHO Strategic Partnership Portal from 2016 to 2019. The JEE score ranges from 1 (no capacity) to 5 (substantial capacity) across 48 indicators. These indicators are divided into four sections: prevention, detection, response and other areas. We used the average score for each of the four sections and the overall average score of the four sections as the final JEE score^c^.

There was no significant association between potential JEE indicators and COVID-19 mortality outcomes or the influenza and pneumonia mortality rate, except for ratification of 18 international human rights treaties. Countries with greater ratification had a higher COVID-19 infections rate (*β*=0.297, p=0.015) and deaths per million (*β*=0.447, p=0.002) (Table 4). This suggests that while greater ratification of human rights treaties is generally positive, it may be correlated with higher COVID-19 infection rates, possibly due to factors like increased international travel or more open borders. Additionally, the influenza and pneumonia mortality rate was positively associated with civil society participation (*β*=0.274, p=0.002) (Table 4). This indicates that higher civil society participation, which often correlates with greater public engagement and transparency, might also reflect higher reporting accuracy or different public health dynamics in those regions.

**Table 4.** Association between COVID-19 and influenza and pneumonia performance and potential JEE indicators across 96 countries

		COVID-19 fatality rate^a^		Number of COVID-19 infections per million^b^		Number of COVID-19 deaths per million^a^		Influenza and pneumonia mortality rate per 100 000 ^a^	
	Potential JEE indicators	*β*	p-value	*β*	p-value	*β*	p-value	*β*	p-value
1. Globalization	Trade	−0.033	0.802	−0.005	0.968	−0.013	0.919	0.004	0.969
2. Variables on high-income countries	Median per capita income	−0.336	0.118	0.063	0.656	0.034	0.874	−0.049	0.742
	Economic freedom (score out of 10)	−0.085	0.645	0.057	0.682	−0.030	0.875	−0.020	0.875
	Civil society participation	−0.001	0.995	0.046	0.714	0.072	0.675	0.274*	0.022
	Ratification of 18 International Human Rights Treaties	−0.096	0.529	0.297*	0.015	0.447*	0.002	−0.155	0.151
3. Health system capacity	Current health expenditure (% of GDP)	0.047	0.718	−0.098	0.373	−0.144	0.275	−0.014	0.879
	Domestic general government health expenditure as percentage of general government expenditure	−0.211	0.177	0.105	0.412	0.239	0.161	−0.189	0.089
4. Political leadership	Trust in government	−0.272	0.348	−0.468	0.104	−0.410	0.210	−0.368*	0.023
	Trust in government (confidence in police total)	−0.344	0.494	−0.022	0.951	0.109	0.865	−0.501	0.082
	Trust in government (trust worthiness and confidence)	−0.169	0.289	0.098	0.444	0.034	0.837	−0.045	0.697
	Trust in government (public trust in politicians)	−0.195	0.184	0.030	0.810	−0.013	0.932	−0.193	0.059
5. Context overlooked	Political stability index (by the Global Economy)	−0.065	0.761	−0.119	0.495	−0.182	0.427	−0.065	0.678
6. Degree of national wealth	Financial globalization indices	−0.225	0.284	0.020	0.909	0.223	0.317	0.002	0.989
7. Equalities	Growth rates of household expenditure or income per capita	−0.155	0.475	0.036	0.834	−0.147	0.523	0.002	0.986
	Proportion of people living below 50% of median income	−0.008	0.952	−0.038	0.747	0.061	0.662	0.087	0.337
	Proportion of population reporting having felt discriminated against, on the grounds of discrimination, gender and disability	−0.357	0.294	−0.322	0.365	−0.250	0.563	0.106	0.447
8. Social security	Social security rate	−0.054	0.769	0.125	0.420	0.035	0.849	0.155	0.201
9. Civil society capacity	2019 Civil Society Organization Sustainability Index	0.273	0.233	0.003	0.984	0.218	0.347	−0.171	0.296

COVID-19: coronavirus disease 2019; GDP: gross domestic product; JEE: Joint External Evaluation.

*p<0.05.

^a^Adjusted population aged 65 y and older (% of total population) and urbanization rate.

^b^Adjusted urbanization rate. For COVID-19 performance, the number of cases (cumulative count of detected and laboratory-confirmed positive cases), the number of deaths (cumulative number of deaths among detected cases) and the number of deaths per 1 million population were extracted from Worldometer as of 9 July 2021. The COVID-19 fatality rate was calculated as the proportion of the number of deaths out of the number of cases. The number of COVID-19 infections per million was calculated as the number of cases per population in millions. The number of COVID-19 deaths per million was calculated as the number of deaths per population in millions. For potential JEE indicators, we collected the data in 2021 from the official websites of international organizations such as the United Nations, World Bank, United Nations Human Rights Office of the High Commissioner, Organization for Economic Cooperation and Development, Sustainable Development Solution Network, the Global Economy, Trading Economics, Family Health International, United States Agency for International Development, International Center for Not-for-profit Law and World Factbook Urbanization.

SDGs 2 (zero hunger), 4 (quality education) and 8 (decent work and economic growth) were inversely associated with the COVID-19 fatality rate (Table 5). Out of 17 SDGs, good health and well-being showed the strongest negative association (*β*=−0.656, p*<*0.001) with the influenza and pneumonia mortality rate (Table 5). The UHC indicators were not strongly associated with COVID-19 performance. However, the influenza and pneumonia mortality rate was negatively associated (*β*=−0.441, p=0.003) with 3.8 UHC service coverage index (Table 5).

**Table 5.** Association between COVID-19 and influenza and pneumonia performances and SDGs and UHC indicators across 96 countries

	COVID-19 fatality rate^a^		Number of COVID-19 infections per million^b^		Number of COVID-19 deaths per million^a^		Influenza and pneumonia mortality rate per 100 000^b^	
SDGs and UHC indicators	*β*	p-value	*β*	p-value	*β*	*P-value*	*β*	p-value
**Overall SDGs (Global Index Score)**	−0.504	0.060	0.088	0.584	0.093	0.692	−0.451*	0.021
**Mean of overall SDGs except SDGs 3**	−0.490	0.064	0.070	0.656	0.120	0.601	−0.378	0.051
Goal 1. No poverty	−0.172	0.335	−0.057	0.652	−0.132	0.392	−0.148	0.258
Goal 2. Zero hunger	−0.386*	0.030	0.144	0.233	−0.312	0.075	0.034	0.797
Goal 3. Good health and well-being	−0.335	0.157	0.181	0.253	−0.136	0.502	−0.656***	<0.001
Goal 4. Quality education	−0.510*	0.015	0.113	0.415	0.013	0.944	−0.319*	0.037
Goal 5. Gender equality	−0.087	0.609	0.069	0.496	0.172	0.233	−0.187	0.125
Goal 6. Clean water and sanitation	−0.076	0.681	−0.127	0.193	0.004	0.978	0.190	0.154
Goal 7. Affordable and clean energy	−0.134	0.576	0.148	0.349	−0.022	0.914	−0.579***	<0.001
Goal 8. Decent work and economic growth	−0.430*	0.007	−0.079	0.464	−0.430*	0.007	0.050	0.677
Goal 9. Industry, innovation and infrastructure	−0.110	0.560	0.006	0.962	−0.183	0.252	−0.111	0.417
Goal 10. Reduce inequalities	0.006	0.964	0.061	0.511	0.041	0.721	−0.098	0.305
Goal 11. Sustainable cities and communities	−0.121	0.507	−0.023	0.838	0.024	0.877	−0.301*	0.022
Goal 12. Responsible consumption and production	0.027	0.859	−0.010	0.926	0.128	0.325	0.032	0.774
Goal 13. Climate action	−0.066	0.613	0.029	0.752	0.206	0.060	0.151	0.104
Goal 14. Life below water	0.197	0.117	−0.053	0.557	0.019	0.864	−0.128	0.161
Goal 15. Life on land	−0.168	0.181	0.148	0.102	0.233*	0.028	0.188*	0.036
Goal 16. Peace, justice and strong institutions	−0.307	0.059	0.044	0.701	−0.125	0.375	−0.187	0.117
Goal 17. Partnerships for the goals	0.305*	0.013	−0.043	0.630	0.098	0.359	−0.023	0.802
3.8. UHC service coverage index	−0.092	0.659	0.067	0.654	−0.101	0.600	−0.441*	0.003
3.8. Catastrophic health expenditure (exceeding 10% of total consumption expenditure)	0.060	0.721	0.009	0.948	0.020	0.904	0.285*	0.025
3.8. Catastrophic health expenditure (exceeding 25% of total consumption expenditure)	−0.019	0.911	0.027	0.840	0.022	0.892	0.317*	0.012

COVID-19: coronavirus disease 2019; SDG: Sustainable Development Goal; UHC: Universal Health Coverage.

*p<0.05.

***p<0.001.

^a^Adjusted population aged 65 and older (% of total population) and urbanization rate.

^b^Adjusted urbanization rate. For COVID-19 performance, the number of cases (cumulative count of detected and laboratory-confirmed positive cases), the number of deaths (cumulative number of deaths among detected cases) and the number of deaths per 1 million population were extracted from Worldometer as of 9 July 2021. The COVID-19 fatality rate was calculated as the proportion of the number of deaths out of the number of cases. The number of COVID-19 infections per million was calculated as the number of cases per population in millions. The number of COVID-19 deaths per million was calculated as the number of deaths per population in millions. The SDGs and UHC indicators were collected from the Sustainable Development Solution Network and United Nations.

## Discussion

We found an inverse association between JEE scores and COVID-19 performance across 96 countries. Specifically, as the JEE scores increased, the number of COVID-19 infections and deaths per million also increased. However, JEE scores were negatively associated with other infectious diseases performance. The influenza and pneumonia mortality rate decreased as the JEE scores increased. This apparent inconsistency may be explained by the differing nature of COVID-19 compared with other infectious diseases. For instance, countries with higher JEE scores may have more robust surveillance and reporting systems, leading to higher recorded cases of COVID-19 due to more extensive testing and detection efforts. By contrast, for more established infectious diseases like influenza, these countries might already have effective prevention and control measures in place, resulting in lower mortality rates. This difference underscores the complexity of pandemic response, where preparedness and health system capacity can result in varying outcomes depending on the disease in question. These findings suggest that while the JEE tool might be effective in predicting outcomes for some infectious diseases, it was less effective for COVID-19, a novel and rapidly spreading pandemic. These results align with findings from similar studies, which have noted the limitations of current health security indices in accurately predicting COVID-19 outcomes.^14^ As for potential JEE indicators, SDGs 2, 4 and 8 were found to be strongly associated with COVID-19 outcomes.

In our study, national health security capacities measured by the JEE did not predict COVID-19 performance in 96 countries, even after adjusting for the proportion of aging population and urbanization rate per country. Similar results were found by Haider et al., where the JEE showed low predictive power value for detection response time and mortality outcome due to COVID-19 in 2020.^7^ It is difficult to conclude the exact reason for this poor result, but perhaps COVID-19 is unique from other infectious diseases.^15,16^ Globalization and an interconnected social and economic world have exacerbated the spread of coronavirus, despite seemingly good preparedness.^17^ The JEE might have underestimated these factors; also, the recent increase in air travel has exacerbated the spread of coronavirus faster than any infectious diseases in recent history.

Meanwhile, the influenza and pneumonia mortality rate, TB incidence and malaria incidence were negatively associated with JEE scores. As the scores increased, these infectious diseases outcomes decreased. This suggests that the JEE score could be an important indicator of national health security against public health threats other than COVID-19. This may be because the JEE was first introduced in 2016, during the pre-COVID era, and was not designed to account for the unique challenges posed by a pandemic like COVID-19. Thus, it might be time to revise the JEE tool to reflect both newly emerged and existing public health crises.

Adding to this discourse, recent research has shown a significant prevalence of COVID-19 among patients with pre-existing chronic conditions such as chronic obstructive pulmonary disease (COPD) and TB. These findings indicate that the burden of pre-existing diseases can exacerbate the impact of COVID-19, suggesting that the JEE tool may need to be adjusted to account for these vulnerabilities. The study highlighted that countries with higher incidences of chronic respiratory conditions might experience worse COVID-19 outcomes, regardless of their general health preparedness, as measured by the JEE.^14^ This is further supported by a study conducted in Punjab, Pakistan, which found that COPD significantly increased the risk of severe outcomes in COVID-19 patients, emphasizing the need for more tailored health security measures in countries with high rates of chronic respiratory diseases.^18^

To propose a revised JEE tool, we examined several potential global indicators such as the SDGs and index. A greater number of these indicators were negatively associated with the influenza and pneumonia mortality rate than COVID-19 outcomes. The higher the trust in government, the lower the influenza and pneumonia mortality rate per 100 000 people. A similar result was observed for SDG 3 (good health and well-being). SDGs 2, 4 and 8 showed an inverse association with the COVID-19 fatality rate. Thus more studies are needed to examine the association of these global indicators with COVID-19 and other infectious diseases performance to propose a revised JEE with a stronger predictive power for infectious diseases.

The Global Health Security Index (GHSI) is another well-known index for health security. The JEE and GHSI are strongly correlated with each other; countries with a high GHSI produced high JEE scores. Nevertheless, a high discrepancy was observed between the GHSI and COVID-19 responses. Haider et al. recommended that the GHSI and JEE scoring tools should embrace additional parameters to better estimate actual pandemic preparedness and vulnerabilities.^7^ A study conducted by Baum et al. reported a poor predictive value of the GHSI to COVID-19 response across 195 countries.^3^ Their study also listed possible factors why the GHSI did not predict COVID-19 performance and provided guidance for the development of a new index on preparedness. Poor performance was also reported across 38 member countries of the Organisation for Economic Co-operation and Development (OECD) during the pandemic.^19^ While OECD countries with higher GHSI scores were expected to be associated with lower COVID-19 outcomes, the opposite result indicated the complexity of each country's response to epidemics and biases resulting from the limited accuracy of GHSI predictions for specific countries.^3^ Such poor results of the GHSI in predicting the COVID-19 performance imply that the GHSI might have underestimated the preparedness of some countries, while overestimating this measure for others.^19^ A counterintuitive correlation between the GHSI ranking and the actual response of countries based on the COVID-19 performance highlights the lack of utility of the GHSI in predicting the response of countries to the COVID-19 pandemic and its impact on them.

Further research is needed to assess the difference in the impact of national public health crisis response capabilities measured at the national level and the JEE between the performance of the response to locally prevalent endemic infectious diseases and the performance of the response to internationally prevalent (truly) pandemic infectious diseases like COVID-19. Also, more studies need to specify the infection route-specific preparation for revising JEE for better or universal working in the future. It would be better to accept inevitable imperfectness to establish accurate prediction or preparedness by extrapolating study results from the previously experienced outbreak only.

This study includes several limitations. First, there is possibility of selection bias regarding the 96 countries included in this study. Countries that are better prepared for public health crises may have been more likely to receive JEE assessments, potentially leading to an overestimation of their JEE scores. Conversely, JEE scores for developing countries, particularly those supported by the USA and intergovernmental organizations, might have been underestimated.^20^ However, it is difficult to expect that these possibilities of biased measurements were associated with outcome variables. Second, there may be biases in data collection related to the JEE process itself. Although the JEE is designed as an objective assessment, differences in how the data were collected, reported and interpreted across different countries could introduce variability that affects the comparability of JEE scores. Third, it is possible that the JEE did not detect associations due to non-differential misclassification with information bias. This type of misclassification could occur if the JEE assessments were not uniformly applied or if there were discrepancies in how countries interpreted the JEE indicators. Although the JEE is an objective assessment, non-differential misclassification might still have occurred. Additionally, external factors such as political stability, economic conditions and healthcare infrastructure were not fully accounted for in this analysis, which could have influenced the association between JEE scores and COVID-19 outcomes. Finally, if COVID-19 incidence is measured based only on clinical symptoms, this could lead to an underestimation of cases, and the number of deaths due to COVID-19 might be overestimated. Therefore, the case fatality rate could have been overestimated because the population age was not standardized.

## Conclusion

The recent literature on an association between the GHSI and COVID-19 performance has been dominated by a focus on the JEE's perceived incompetence and alleged uselessness. However, what stands out most from our study is that JEE scores were effective in explaining the outcomes of most infectious diseases, including pandemic influenza. Given the JEE tool is the fundamental component of the IHR Monitoring and Evaluation Framework^20^ and that the JEE scores reflect IHR core capacity implementation, it is difficult to conclude that the IHR (2005) has not functioned properly.^21^ Moreover, it would be a serious oversight to conclude that the JEE tool does not properly assess a country's ability to deal with public health crisis capabilities and that the JEE process is ineffective. The JEE has been a reliable tool for assessing infectious diseases, although it may not have been as predictive for COVID-19.

To improve readiness for pandemics similar to COVID-19, factors affecting the performance of responses to pandemic infectious diseases, such as COVID-19, should be identified and integrated into the JEE tool through additional research. Future research should specifically focus on enhancing the JEE tool to account for the unique challenges posed by rapidly spreading pandemics, including the impact of globalization, healthcare system resilience and the integration of real-time data analytics for better predictive capabilities.

## Data Availability

The data underlying this article will be shared on reasonable request to the corresponding author. Publicly available data were used in the study. All data relevant to the study are provided with the sources in the article.
